# Unusual Origin of the Anterior Scrotal Nerve: A Case Report

**DOI:** 10.7759/cureus.4557

**Published:** 2019-04-27

**Authors:** Karishma Mehta, Joe Iwanaga, R. Shane Tubbs

**Affiliations:** 1 Clinical Anatomy, Seattle Science Foundation, Seattle, USA; 2 Medical Education and Simulation, Seattle Science Foundation, Seattle, USA; 3 Neurosurgery, Seattle Science Foundation, Seattle, USA

**Keywords:** scrotum, femoral nerve, ilioinguinal nerve, anatomy, cadaver

## Abstract

The anterior scrotal nerve is a cutaneous nerve that branches from the ilioinguinal nerve after it leaves the superficial inguinal ring. However, we identified a cadaveric specimen with an anterior scrotal nerve arising from both the femoral and ilioinguinal nerves. This anatomic variation should be considered with anesthetic blockade of this region or during surgical procedures nearby.

## Introduction

The anterior scrotal nerve provides cutaneous innervation to a portion of the penis and the upper scrotum in males. It arises from the ilioinguinal nerve after the aforementioned nerve passes through the superficial inguinal ring [1]. Both the ilioinguinal and femoral nerves originate from the lumbar plexus via ventral rami of L1 and L2-4, respectively. Normally, the femoral nerve gives rise to two cutaneous branches, anterior femoral nerves, and the saphenous nerve [2-3]. Herein, we report a case in which the anterior scrotal nerve arose from both the ilioinguinal and femoral nerves.

## Case presentation

**Figure 1:**
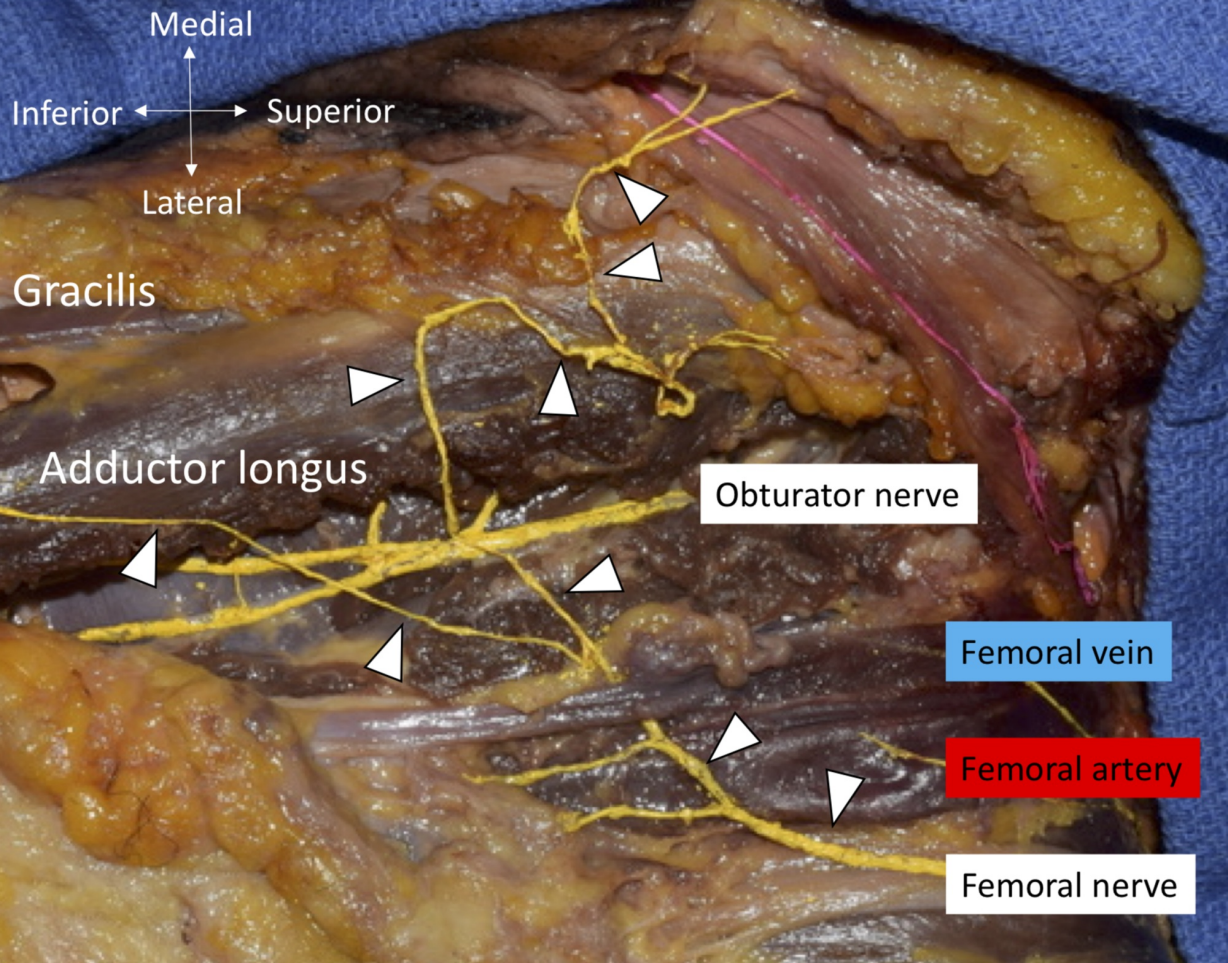
MFC nerve of the thigh (arrowheads) and obturator nerve are colored yellow Note one of the branches of the MFC travels medially to innervate the skin of the anterior scrotal area. MFC, medial femoral cutaneous

**Figure 2:**
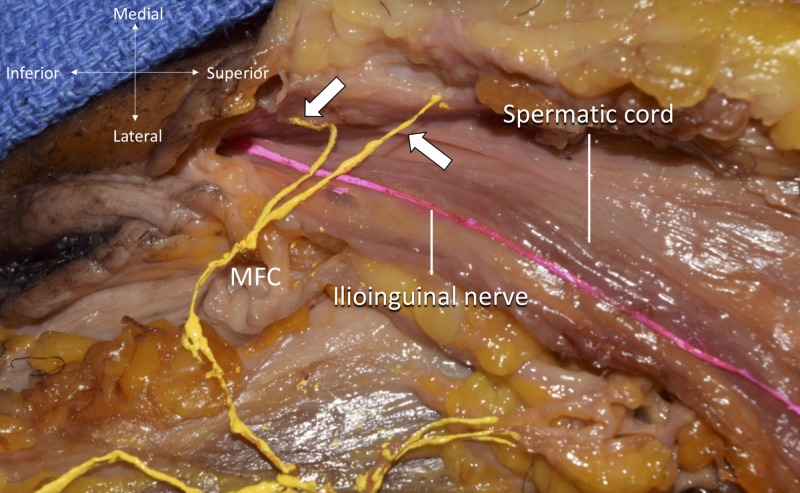
Magnified photo of previous image Note two anterior scrotal branches (arrows) were given off from the MFC nerve. MFC, medial femoral cutaneous

During routine dissection of the thigh, a variant anterior scrotal branch was found in an African-American fresh-frozen male cadaver whose age at death was 79-years-old. The anterior division of the femoral nerve gave rise to two cutaneous nerves, the medial femoral cutaneous nerve of the thigh (MFC) and the intermediate cutaneous nerve of the thigh (ICN). The MFC was traced and found to supply three branches to the skin of the anterior and medial thigh. The MFC then traveled medially and superiorly to join the anterior scrotal branch of the ilioinguinal nerve which coursed superficial to the spermatic cord (Figures 1, 2). The origin of the femoral nerve and ilioinguinal nerves was L2-4 and L1, respectively. There were no variations of the iliohypogastric or genitofemoral nerves.

## Discussion

Although prior anatomical studies have been performed with regard to the femoral and ilioinguinal nerve branches, none have focused on the origin of the anterior scrotal nerve. Gustafson et al. dissected femoral nerves and observed alternate branching patterns (i.e., order, medial to lateral location) of its cutaneous subdivisions [4]. Rab et al. noted anatomical variations of the cutaneous contributions from the ilioinguinal and genitofemoral nerves. Of note, “type D” stated that in 7.8% of their cadavers, both the ilioinguinal and genitofemoral nerves supplied innervation to the scrotal area, bilaterally [5]. Iwanaga et al. revisited the anatomy of the genitofemoral nerve and suggested a new terminology for the branches of the genitofemoral nerve based on its proximal course i.e., medial and lateral branches [6]. Also, the ilioinguinal nerve can be replaced by the iliohypogastric nerve or the genital or femoral branches of the genitofemoral nerve [7]. Thus, the innervation of the superior portion of the anterior thigh and genital area might necessitate remapping in regard to their cutaneous supply. Despite these prior findings, to our knowledge, a case similar to ours has not been reported.

## Conclusions

The anterior scrotal nerve has historically originated from the ilioinguinal nerve; however, we report a unique case study in which the anterior scrotal nerve originates from both the ilioinguinal and femoral nerves. This finding is significant as it provides insight for surgeons and anesthetists alike, especially when performing procedures such as cutaneous nerve blocks.
